# 2674. Disparities in Mpox Care: Use of the Social Vulnerability Index to Predict Mpox Infections

**DOI:** 10.1093/ofid/ofad500.2285

**Published:** 2023-11-27

**Authors:** Michael Leonard, Robert Fairman, Christopher Polk, Michael Inman, Catherine Passaretti, Mindy Sampson

**Affiliations:** Carolinas Medical Center Atrium Health, Charlotte, North Carolina; Tulane School of Medicine, Charlotte, North Carolina; Atrium Health, Charlotte, North Carolina; Atrium Health/Wake Forest School of Medicine, charlotte, North Carolina; Advocate Health, Charlotte, NC; Atrium Health, Charlotte, North Carolina

## Abstract

**Background:**

The 2022 mpox outbreak disproportionately impacted minoritized populations especially in the US South, where 60% of infections were in Black men. Social vulnerability may explain some of the disproportionate disease burden as minoritized populations are at higher risk for infections such as mpox. The goal of this study is to examine what social vulnerabilities predict mpox infection.

**Methods:**

We performed a retrospective chart review of all patients tested for mpox between July 24 and September 28, 2022 within the largest healthcare system in the Carolinas. Demographic information, medical and sexual history, and patient geocoding was obtained from extracted data. Patient location was then joined to the CDC and Agency for Toxic Substances and Disease Registry Social Vulnerability Index (SVI) data at a census tract level. Logistic regression models predicting mpox test results were then constructed.

**Results:**

Of 319 individuals tested for mpox during the study period, those who identified as Black, men who have sex with men, having multiple sexual partners, and those known to be living with HIV were more likely to test positive for mpox (p< 0.001, see Table 1). In assessing the social vulnerability sums, there were statistically significant differences in the household composition and disability sum of those who tested positive for mpox (mean=1.6952, std=.0663) and those who did not (mean=1.8495, std=.5712, p=.0349), as well as a statistically significant difference in the means of minority status and language sum (p=.0001). Being in a census tract that is 90^th^ percentile for multi-unit housing (p=.0003), as well as a census tract that is in the 90^th^ percentile for unemployment (p=.0393) are associated with testing positive for mpox, but overall SVI was not predictive of mpox diagnosis (see Table 2).

**Figure:**
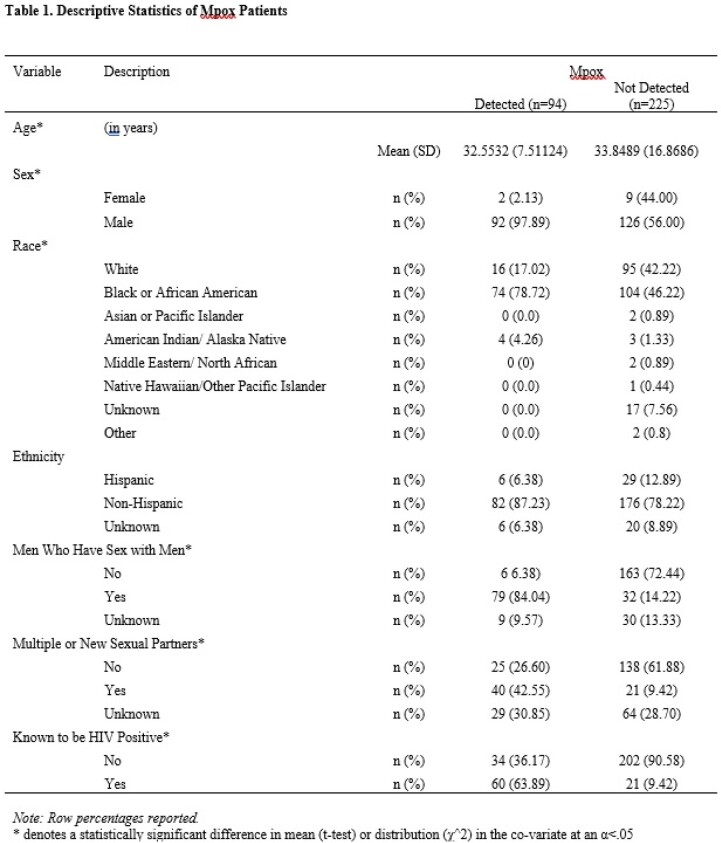

**Figure:**
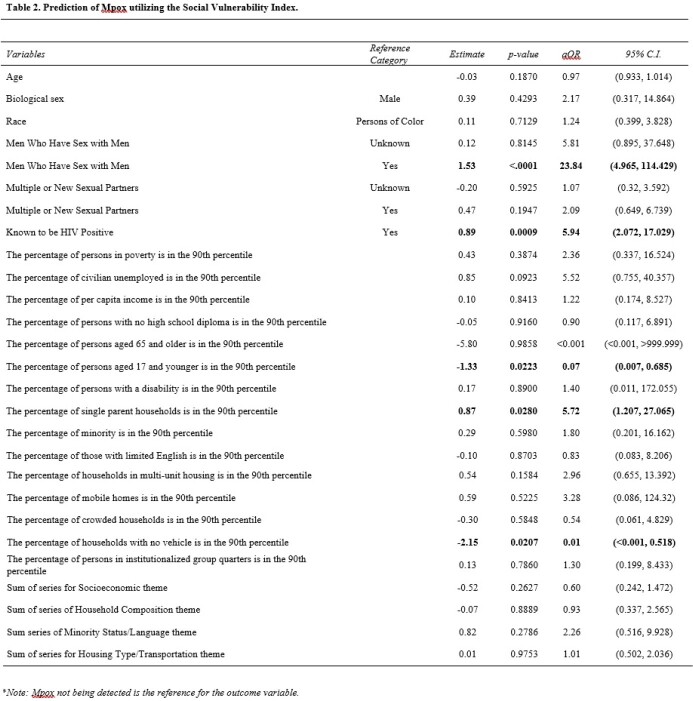

**Conclusion:**

Being diagnosed with mpox primary occurred among those who are Black, men who have sex with men, and those living with HIV. Though some social vulnerabilities predicted mpox diagnoses, overall, social vulnerability did not predict mpox diagnoses.

**Disclosures:**

**Christopher Polk, MD**, ViiVHealthcare: Job change to work for ViiV as Medical Director

